# Update on the Epigenomic Implication of Embryo Cryopreservation Methods Applied in Assisted Reproductive Technologies With Potential Long-Term Health Effects

**DOI:** 10.3389/fcell.2022.881550

**Published:** 2022-04-28

**Authors:** Arturo Reyes Palomares, Kenny A. Rodriguez-Wallberg

**Affiliations:** ^1^ Laboratory of Translational Fertility Preservation, Department of Oncology and Pathology, Karolinska Institutet, Stockholm, Sweden; ^2^ Division of Gynecology and Reproduction, Department of Reproductive Medicine, Karolinska University Hospital, Stockholm, Sweden

**Keywords:** epigenetics, ZGA, cryopreservation, single-cell multi-omic, embryo, long-term effect, ART

## Abstract

Cryopreservation of embryos has become an efficient method in Assisted Reproductive Technologies (ART) and these methods are currently performed at nearly all fertility centers around the globe. Cryopreservation of supernumerary embryos has contributed to an increase in cumulative pregnancy rates and as a consequence, an increasing number of children are being born through these techniques worldwide. However, long-term follow-up studies of children born through ART are scarce, and concerns about the long-term health effects on individuals conceived through ART have been raised. The relevant genomic transformations that occur at the time cryopreservation is usually applied to embryos may have potential epigenetic risks. With advances in multi-omic single cell technologies, new ways to assess the (epi)genomic status during early embryo development have now become feasible. These novel strategies could provide a revolutionary opportunity to understand the actual impact of ART, but also may help future developments aiming at increase both their efficiency and safety. Here we outline insights in current knowledge and research on the impact of cryopreservation on embryos, the possible consequences at epigenetic level and how emerging new high-throughput technologies can be used for their assessment.

## Introduction

Since the birth of the first baby conceived through by *In Vitro* Fertilization (IVF) in 1978, success of Assisted Reproduction Technologies (ART) has greatly increased. This has been achieved through continuous improvements in culture methods, sperm preparation, fertilization techniques and ovarian stimulation protocols that gradually yielded improved outcomes in terms of pregnancy and live birth rates. More than 3.3 million treatments are performed every year and to date it has been estimated that 10 million children have been born through ART worldwide (ESHRE European Society of Human Reproduction and Embryology; De Geyter et al., 2020; Adamson, 2021). The IVF process mimics and also bypasses several physiological reproductive paths and it inherently involves a variable degree of invasiveness with unknown consequences. The IVF encompasses hormonally controlled ovarian hyperstimulation, interventional oocyte retrieval, oocyte denudation at the laboratory, insemination of the oocytes through conventional IVF or intra-cytoplasmic sperm injection, culturing of embryos for extended time and in many cases cryopreservation and thawing of supernumerary embryos. All these procedures are performed closely or within the frame time for the nascent genome assembly.

How ART could impact over the health of the children born through these different technologies is still unclear and there is a growing concern in the field. Worldwide efforts for improve surveillance are progressing, and several countries started to systematically collect health population data in registries, which enables to follow large ART cohort populations. One of the main interests has been the assessment of long-term effects induced by ART. However, still the gross of this population is very young and enacting an assumption can be premature. Epidemiological studies performed up to date in large cohorts have shown a higher incidence of childhood cancer associated to IVF in an American population cohort of 275 686 ART conceived vs 2 266 847 naturally conceived children (Spector et al., 2019). Also, a Danish population study observed an increased risk of childhood cancer after frozen/thawed embryo transfer (FET) in a population of 174 881 children conceived through ART compared to 910 291 naturally conceived (Hargreave et al., 2019). A large epidemiological study performed by our group using the Swedish population-based registers, compared 43 506 children born after ART vs 2 847 108 naturally conceived children and found a significantly increased risk of infant mortality from birth up to 1 year of life in singletons after ART, specifically when FETs were applied (Rodriguez-Wallberg et al., 2020). High birthweight and large children for gestational age have been associated exclusively with FET in previous European studies (Magnusson et al., 2021; Westvik-Johari et al., 2021). All these findings have been identified early in life and the long-term consequences when the ART population becomes older are unknown. A recognized limitation in all studies is the confounding impact of the underlying cause of infertility of the biological parents in the obstetrical and perinatal complications, but also the diversity of techniques applied. A known high-risk factor is the multiple conception and multiple births, often confounding factors in epidemiological studies attempting to explore health disorders related to ART. With this regard, controlled experimental designs would ideally provide meaningful insights about the real impact of ART and the emergence of related hypothetical disorders. The objective of this review is to overview currently used cryopreservation methods and trends in ART and discuss the concerns raised about their potential impact on embryos at the light of the recent knowledge about the regulation of embryo genome.

### Cryopreservation Is a Clinically Established Practice by Necessity but With Still Uncertain Impact

Great efforts have been made for improving cryopreservation methods in reproductive medicine up to a clinical integral path within the IVF processes. A large sequence of protocols evolved to reach an optimized combination of permeating and non-permeating cryoprotectants (CPA) for reducing ice formation, maintain osmolarity and subsequently preserve structure and viability of cells (Rodriguez-Wallberg et al., 2019; Whaley et al., 2021). Cryopreservation was mostly based on slow freezing protocols in the beginning and still today several of these protocols are preferred for cryopreservation of sperm cells, testicular tissue and ovarian cortex. The emergency of vitrification supposed a reliable and robust protocol that highly improved survival rates of embryo cryopreservation (Kuwayama et al., 2005; Kuwayama, 2007). The good results obtained with vitrification made easy to implement this method in IVF laboratories worldwide for cleavage and blastocyst embryos, but also for the challenging oocytes (Rienzi et al., 2017). Actually, embryo cryopreservation is required for accomplish with current guidelines in many countries in order to follow safety policies, i.e. the practice of single embryo transfer (SET) recommended to reduce adverse perinatal outcomes inherent of multiple pregnancies, which usually requires an efficient embryo cryopreservation program (Tiitinen et al., 2001). The application of Pre-implantation Genetic Testing through biopsies at blastocyst stage also commonly require their vitrification while awaiting for the results of genetic analyses (Carvalho et al., 2020). Vitrification of embryos is additionally increasing and being performed to a large extent in clinical practice under the proposed “freeze all” IVF cycles, an strategy intended to reduce potential negative effects of ovarian hyperstimulation that hypothetically could compromise endometrial receptivity in the fresh cycle (Shapiro et al., 2014). Improvements in cancer care are also increasing the demand of cryopreservation methods within programs for fertility preservation (Rodriguez-Wallberg and Oktay, 2014), as well as the recognition of societal and lifestyle factors, gender equality access to fertility treatments and new family models (Homan et al., 2007). During the last 2 decades the number of frozen-thawed embryo cycles has been growing progressively in Europe, United State and Australia, with cryopreserved cycles surpassing the number of fresh cycles in the last two countries according to register data (De Geyter et al., 2020). This trend is expected to largely increase the proportion of children born via cryopreservation in ART conceived children. Despite this increasing number of frozen-thawed embryo cycles, a lower live birth rates have been recently reported compared to fresh when using in both cases fresh donated oocytes in the origin (Insogna et al., 2021), which indicates that cryopreservation may inherently impact over the embryo and this could be reflected in treatment success.

Current vitrification combines CPAs such as dimethyl sulfoxide (DMSO), Ethylene glycol (EG) and sucrose or trehalose, during a timely controlled exposure and the cooling and warming rates are controlled through the sample volume (Kuwayama, 2007; Chang et al., 2022). The direct toxicity of the most commonly used CPAs is a potential source of damage with unknown consequences. DMSO and EG can induce methylation and structural changes in the chromatin. The DMSO can also facilitate DNA double-strand breaks (Nelson and Curtis Johnson, 1970). The damage induced by CPAs is dependent on concentration, pH fluctuation, temperature, degree of hyperosmotic stress and reactive oxygen species (ROS) generation (Kopeika et al., 2015). It is worth to mention that the most widely performed current technique of vitrification drastically increase osmolality up to 20 times more than what is standardized for embryo culture conditions, although osmolality is restored after warming (Chang et al., 2022). During these processes changes in membrane permeability led to a rapid exchange of water influx that modifies the embryonic cell morphology. This transient shrinking and expansion ends up with a forced metabolism arrest once the embryos are plunged into liquid nitrogen. Under optimal conditions, after thawing, all these changes are “visually reverted” but it is worth to remark that this transitory variability in the dynamic of embryo cleavage conveys morphologic differences in cell shape and size of blastomeres, presence of fragments, asymmetry between cells, differences in cell cycle state accounting for many variables that can have a further impact. Cryopreservation impacts directly over the cytoskeleton structure (Chatzimeletiou et al., 2012), inducing changes in shape and size of the blastomeres, which could hypothetically be relevant by impacting over ZGA onset (Hui Chen et al., 2020). Studies of transcriptome and proteome have reported significant changes in human embryonic stem cells and Wharton’s Jelly Stem Cells after cryopreservation (Wagh et al., 2011; di Giuseppe et al., 2014).

### Cryopreservation Could Impact Over Nascent Genome Reorganization and Polarity Establishment in the Pre-implantation Embryo

Fertilization releases the more critical processes required for drawing the maternal-to-zygote transition (MZT) and subsequently zygote genome activation (ZGA). The reprogramming is characterized by a robust initial erasure of epigenomic marks, lasting until totipotency acquisition, which then enables a free way towards self-differentiation. Along further development, the new epigenome is gradually established along the cell lineage establishment creating a new embryonic self-identity (Gretarsson and Hackett, 2020). This sharp demethylation increases chromatin accessibility, but still the transcriptional activity is mostly dependent on the maternal support provided by the oocyte. Histone post-translational modifications H3K4me3 and H3K27me3 are critical regulators of spatiotemporal transcriptional activity showing a concrete pattern during reprogramming. During human early embryo development, H3K4me3 locates at CpG-rich regions of promoters for activating the transcription of genes until the 8-cell stage. A remaining 47% of CpG-rich promoters sites corresponding to differentiation and developmental genes are currently lacking H3K4me3 (Xia et al., 2019). Additionally, a distal distribution of H3K4me3, which is also absent in oocytes, is observed in 4-cell stage embryos, indicating *de novo* deposition after fertilization (Xia et al., 2019). H3K27me3 is progressively lost until ZGA, showing a characteristically slight delay in the maternal genome, and around compaction in morula stage it starts to be deposited again. Intriguingly, later on at blastocyst stage, it will be found asymmetrically distributed among the inner cell mass (ICM) or the trophectoderm (TE) (Xia et al., 2019). The emergency of topological associated domains (TADs) as part of the 3D chromatin structure of early human embryos is a current subject of debate. Despite its biological significance is still unknown, recent studies indicate that TADs consolidation coincide with ZGA (Chen et al., 2019).

**FIGURE 1:**
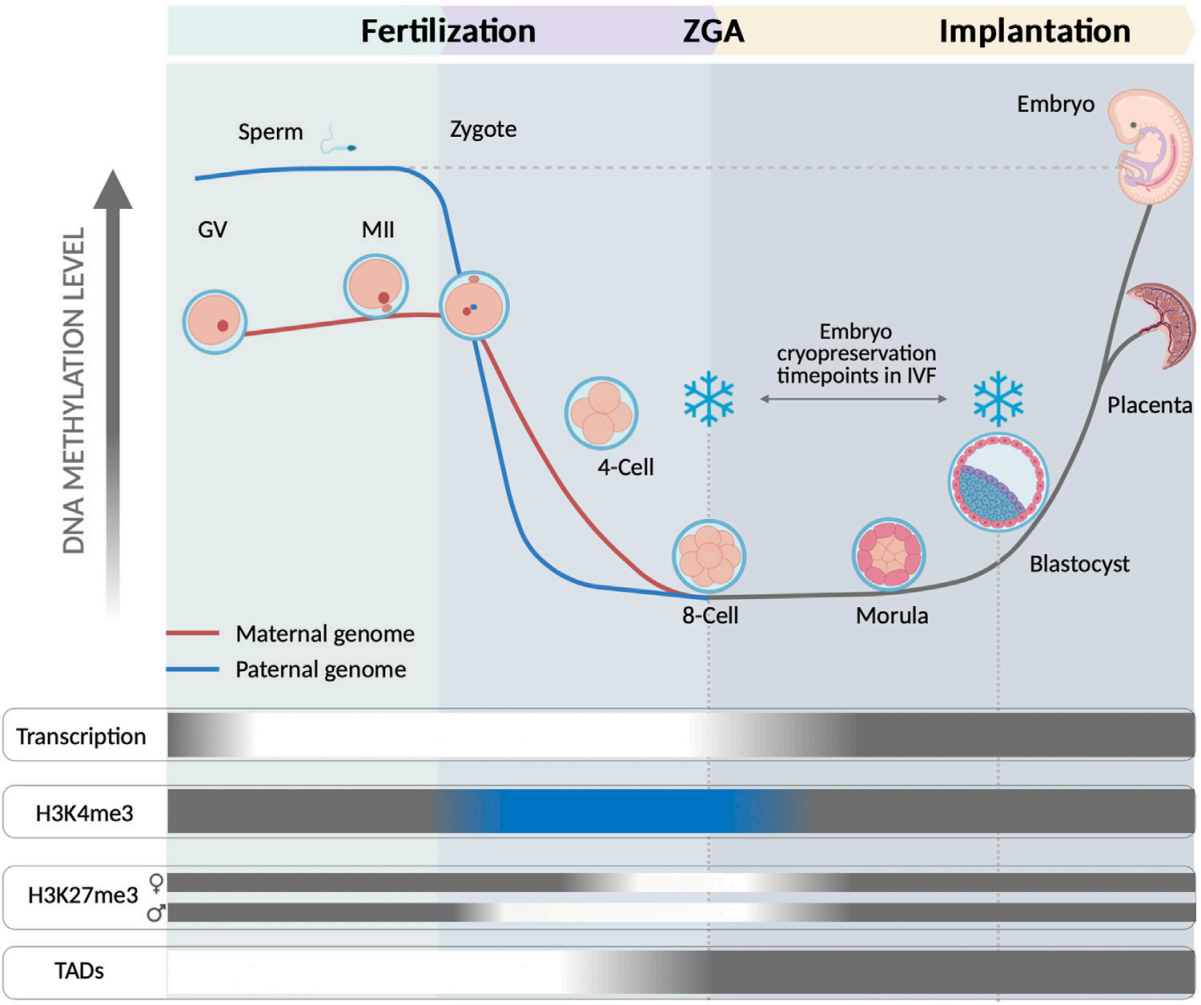
Representation of the human embryo epigenomic state at 8-cell and at blastocyst stage, both common timepoints for application of cryopreservation within clinical ART. Fertilization releases embryo reprogramming by inducing a wide remodeling along different epigenetic layers. The 8-cell stage and the blastocyst stage are illustrated to represent their very different epigenomic states of their embryonic cells. A sharp demethylation takes place in both, the sperm and the oocyte genomes, with this last showing a more paused progression. The demethylation becomes maximum at 8-cell stage, coinciding with the ZGA. BLUE indicates an unusual positioning of at H3K4me3 on CpG rich regions at promoters of transcribed genes and also positioned at distal CpG rich and hypomethylated regions until 8-cell stage. Intriguingly, H3K4me3 lacks at CpG rich promoter regions of developmental and differentiation related genes (Xia et al., 2019). H3K27me3 is progressively erased after fertilization and completely absent during ZGA, with a slight delay in the maternal genome. An asymmetric distribution of H3K27me3 has been shown in blastocyst by its positioning in ICM cells at distal cis-regulatory regions of developmental genes (Xia et al., 2019). TADs formation arise from 8-cell and according to previous observations is dependent on ZGA onset, which surrounds 8-cell stage (Chen et al., 2019). GREY areas show a common pattern observed in differentiated cells; WHITE indicate a loss of activity; BLUE indicates a different role than that of differentiated cells.

**FIGURE 2:**
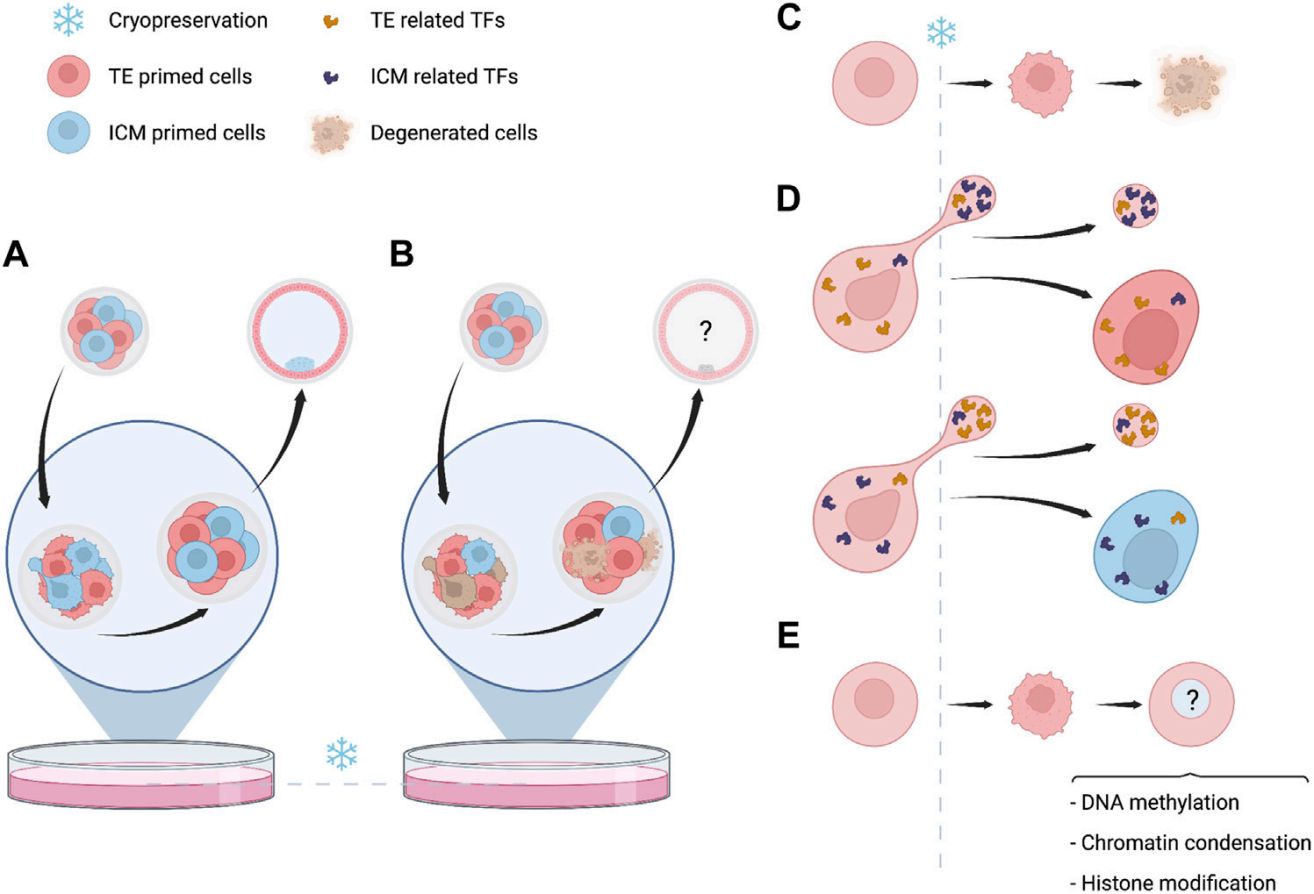
Events related to embryo cryopreservation with potential developmental consequences. **(A)** Full recovery of embryo viability without evident changes in morphology or organization. **(B)** Difference in survival rates between embryonic cells can lead to variation in the proportion of cells primed towards trophectoderm (TE) or inner cell mass (ICM). The question marks refer to unknown consequences for further embryo viability and/or cell lineage establishment. **(C)** Degeneration of cells is a common morphological evidence of damage induced by cryopreservation. If few cells are damaged, embryos still can survive, implant and successfully result in pregnancy and live-birth. The effect of this loss of material is still unknown. **(D)** During cleavage cytoplasmic material is transiently released and subsequently reabsorbed. The causes of this phenomenon are unknown and probably stochastic, also the biological significancy of these projections is unknown. However, if interrupted by cryopreservation, a potential modification of the cytoplasmic content can be hypothetically considered. **(E)** Even if complete survival of blastomeres is reached, which is more common with modern vitrification methods, some changes over different epigenetic layers may still persist and must be considered.

The application of IVF techniques and cryopreservation methods overlap in the time with this wide remodeling of the nascent genome (Figure 1). Exposure of embryos to non-physiological conditions along this entire sensitive period could have a cumulative negative impact (Wale and Gardner, 2016). Under optimal conditions after thawing, the impact of cryopreservation is morphologically re-established, however, the subtle changes at subcellular and molecular level could have further consequences. During the very early stages of embryo cleavage, genomic rearrangements will depend on the initial maternal support provided by the oocytes. This support mostly consists in TFs acting over cis regulatory elements (CREs) and interacting with different epigenomic layers, while the new embryo evolves towards ZGA. Along cleavage progression, an interacting network between different epigenetic layers derive in a primed version of cells that would progress towards TE or ICM (Wu et al., 2018; Wang et al., 2021). Low-input multi-omic analysis have shown that asymmetric distribution of regulatory elements involved in the cell fate may be identified at very early stages of embryo development, before the hypothetical cell lineage segregation (Xia et al., 2019; Wang et al., 2021). The diversity of cells, their cell-cell contact interaction and the content of regulatory elements as metabolites, proteins and TFs, are relevant for further developing a proper cell lineage establishment (Stadhouders et al., 2019). The establishment of cell identity probably depends on the interplay between surrounded environmental conditions stochastic factors and the nuclear components defined in a specific cellular state. A regulatory mechanism associated to ZGA and mediated by AP-2 gamma (Tfap2c) and TEA domain transcription factor 4 (Tead4) has been recently identified. It determines induction of polarization in the embryo and the further fate of cells towards TE or ICM (Zhu et al., 2020). Zhu et al. have proposed that symmetry of cells may be disrupted by effect of two contrary mechanisms leading to concentration of apical proteins by actin-mediated cooperative recruitment of Tfap2c and Tead4, compensated by a diffusion effect of lateral mobility. Such mechanisms are sensitive and could be affected by a direct effect of cryopreservation. In addition, during cleavage, blastomeres extrude and reabsorb fragments and cellular projections (Hardarson et al., 2002), and an asymmetric distribution of mitotic spindle is involved in cell fate determination, which could influence gene expression and chromatin regulators (Pomp et al., 2022). The induction of cryopreservation at a specific timepoint could hypothetically affect these asymmetric transitions and also regulatory elements, affecting the distribution of TF or cytoskeletal components associated to cell lineage establishment (Figure 2). In clinical practice embryo cryopreservation is arbitrarily carried out either at cleavage or at blastocyst stages. Cryopreservation is thus acting on different genome and cellular components in these embryos, corresponding to minor and major waves of ZGA, respectively (Figure 1).

Still there is a lack of knowledge about how methylation is reloaded after thawing, how chromatin structure is restored after drastic changes in the cell structure and how fidelity of transcriptional activity is maintained after interchanges of cytoplasmic components as consequence of water influxes caused by changes in osmotic states. However, the new insights about epigenome regulation and cell fate determination in the early embryo invite to hypothesize that a sudden forced arrest, such as that induced by cryopreservation, could have consequences over these highly dynamic and fine-regulated processes.

### Epigenomic Changes Associated to Cryopreservation of Embryos

The success of cryopreservation lies in the avoidance of damage caused by ice crystal formation, which is controlled through both an equilibrated concentration of cryoprotectants and adapted cooling rates to effectively interchange cytoplasmic and extracellular water. The variability attained through described changes in osmotic and oxidative stress, the potential toxic effects of CPAs, the changes in size and morphology of embryonic cells and on global embryo polarity, can all potentially interfere with genome integrity and functionality and transmit epigenetically inherited marks. During embryo culture the increase of ROS could also have a harmful effect over the embryo culture (Guérin et al., 2001). With this regard, the effect of a lower oxygen concentration during embryo culture has shown to be beneficial, and largely interpreted as by mimicking the Fallopian tubes conditions (Kirkegaard et al., 2013), which has been subsequently and successfully implemented worldwide (Christianson et al., 2014). Cryopreservation could lead to disbalanced ROS and oxidative stress, which has been potentially linked to interfere between CpG sites and transcription factors, but also with aberrant promoter hypermethylation and global hypomethylation (Dattilo et al., 2016; Menezo et al., 2016). At a higher order of organization, in the chromatin, cryopreservation can induce condensation and collapse of replication fork with potential consequences in active cleavage embryos (Falk et al., 2018). A recent study additionally demonstrated that cryoprotectants induce epigenetic changes in cells in culture even at very low concentration (Verheijen et al., 2019).

After widespread demethylation metastable epialleles are established along developmental progression and their variability depend on stochastic factors, periconceptional environment exposures and genomic conditions (Kessler et al., 2018). A recent study reported highly conserved epigenetic signatures shared by monozygotic adult twins, with a higher correlation in monochorionic compared to dichorionic monozygotic twins (van Dongen et al., 2021). These findings suggest that some relevant epigenetic signatures are established very early, even before embryo implantation, and can be maintained through life. Several studies have identified differentially methylated regions in fetal, placenta and cord blood samples of children born after ARTs compared to naturally conceived children (Song et al., 2015; El Hajj et al., 2017; Ghosh et al., 2017; Ye Liu et al., 2021). However, some authors have argued that these differences may be resolved during adulthood, as they did not observe such differences in methylome profiles of adolescents born through ART (Novakovic et al., 2019; Penova-Veselinovic et al., 2021). However, these studies do not discard that other epigenetic variable may be altered. A positive correlation between ART and four imprinting disorders have been also found in a previous systematic review and comprehensive metanalysis study (Cortessis et al., 2018). Studies in bovine models reported a higher variability of differentially methylated regions at imprinted genes during the preimplantation period (O’Doherty et al., 2015). This variability has been corroborated by others studies that hypothesized the existence of active mechanisms that stabilize imprinted genes until blastocyst stage (Ivanova et al., 2020). This could indicate a certain vulnerability of imprinting genes to IVF but also to cryopreservation.

Impact of ART over histone modification has been currently analysed to a lesser extent due to their technical limitations. Recent studies using high-throughput technologies have reported variations in the methylation degree and the histone modification patterns (H3K4me3) in cord blood cells of new-borns and foetuses originated through IVF (Wei Chen et al., 2020; Ye Liu et al., 2021; Yang et al., 2021). Very interestingly, the higher degree of variability was observed in the more invasive techniques as FET and ICSI (Wei Chen et al., 2020). In another study it has been shown that vitrification and thawing decrease acetylation state of histones H3K9ac and H3K27ac in embryo culture (Jahangiri et al., 2018; Truong and Gardner, 2020).

A multi-omic analysis based on cord blood of different IVF techniques, revealed a higher number of differentially expressed genes in children born after FET (Wei Chen et al., 2020; Zongzhi Liu et al., 2021). A study based on mice models reported differences induced by vitrification in transcriptional profile of microRNAs (miRNAs) over biogenesis pathway when cryopreservation was performed during the earlier stages of preimplantation embryos (Azizi et al., 2021); by contrast in later stages, it involves miRNAs related to embryo implantation (Zhao et al., 2015).

Substantial research has been performed in animal models with wide demonstration of epigenetic long-term consequences of reproductive and embryo technology (Zhu et al., 2021). The fundamentals of techniques applied in these studies are similar to the ones applied clinically in humans. Also, this is a good resource for guiding future translational research aimed to optimize and minimize the impact of ART in humans.

### New Single-Cell Multi-Omic Analysis can Open a New Era to Improve Actual Assisted Reproduction Technologies

Up to date the epigenetic impact of ART has been mostly analysed by focusing on one specific genomic epigenetic layer. In another situations, many studies focused in specific candidate gene regions on array-based or PCR technologies, that could underestimate the impact over the whole genome. New approaches based on integrative methodologies at single-cell level become a more adapted strategy to provide a wide landscape (Barberet et al., 2020). The development of new multi-omic strategies based in high-throughput technology and single-cell methods have expand horizons in the field of developmental epigenetics and genome regulation. Data integration of different epigenetic layers provide a very complete snapshot of the global epigenomic diversity of every single-cell conforming the embryo. This application led to new approaches for deciphering the regulatory elements and their mechanistic interactions along genome reorganization during early embryo development (Wu et al., 2016; Guo et al., 2017; Zhu et al., 2017). Similar strategies have been succesfully applied at more advanced developmental stages, to explore regulatory mechanisms modifying the chromatin and involved in cell fate determination (Cusanovich et al., 2018).

Recently single-cell multi-omic technologies have improved efficiency with a very low input of cells, as the case of single-cell nucleosome, methylation and transcription sequencing (scNMT-seq) and single-cell nucleosome occupancy, methylation and RNA expression sequencing (scNOMeREseq) (Clark et al., 2018; Wang et al., 2021). One advantage of these approaches resides in the simultaneous capture of DNA methylation, chromatin accessibility and transcriptomic signals of every single cell of the embryos. This integrative information is very valuable to provide meaningful insights about the regulatory processes representative in every single-cell during different stages of the pre-implantation period. Profiling individual cells increase the predictive potential about the impact of those conditions applied over the embryos, as it is the case of cryopreservation or other IVF methods. The implementation of these technologies for translational research in reproductive medicine could allow research of supernumerary human embryos after IVF treatment donated for research and of human blastoids, as an alternative option.

The integration of spatial and single-cell transcriptomics data has been used to profile genomic expression of organs during early development (Asp et al., 2019); a high-resolution analysis has been used to recapitulate cell-fate decision during development of entire early embryos (Lohoff et al., 2021). The development of spatial transcriptomics, using animal models, could serve as an excellent strategy for exploring persisting functional alterations induced by cryopreservation or other IVF conditions in target tissues of embryos at further developmental stages.

## Concluding Remarks

Cryopreservation has become essential in clinical reproductive medicine and it is widely applied at IVF units worldwide. The continuous increase in the number of children born after cryopreservation and the lack of knowledge regarding their long-term consequences is a growing concern in the field. The recently developed high-throughput technologies at single-cell level are providing meaningful insights about early embryo biology. As discussed in this review, these cutting-edge technologies have shown the possibility to simultaneously analyse different epigenetic layers at one single cell level what can be applied to widely assess the impact of current methods applied in ART. In addition, these multi-omic approaches can unveil unknown details about epigenetic alterations in the preimplantation embryo, bringing a new tool for validating of the current IVF methods and could also potentially contribute to their further development. Some limitations arise in the application of these techniques, especially those concerning the capture of appropriate signals that truly represent the intended resolution, but fortunately their application is continuously being developed and improved. The design of studies based on supernumerary embryos of IVF cycles can contribute to gain an essential knowledge to increase surveillance of children born after ART. Under a more comprehensive assessment, in animal models, these technologies applied in the early embryo combined with new emerging high-resolution spatial omics, could provide mechanistic insights as well as a wide and measurable landscape about the impact of ART with downstream consequences. Nowadays there exists a higher tendency towards cryopreserving embryos at blastocyst stage, but still there is a lack of knowledge about which is the more convenient timepoint for embryo cryopreservation considering health consequences. The morphological embryo assessment is the most common criteria up to date; however, it should not be disregarded that an epigenetic criterion could potentially complement future decisions concerning a safer prognosis in terms of impact. ART are highly demanded and very often the unique path to accomplish reproductive parental projects in our modern societies. The application of these novel technologies in research arises as a good opportunity to reduce uncertainties concerning the health of children conceived through ART.
